# Supplementary material for: Comparative mitogenomic analyses and gene rearrangements reject the alleged polyphyly of a bivalve genus
Source: PeerJ. 2022 Sep 26;10:e13953. doi: 10.7717/peerj.13953 (PMC9521344; doi:10.7717/peerj.13953)
Supplement: Supplemental Information 1 — Gene rearrangement scenarios from the ancestral gene order of Musculista senhousia to the gene order of Perna viridis identified by the CREx analysis. Family diagrams represent the observed gene order and blocks of inferred rearrangement events highlighted in blue representing tandem-duplication-random-loss (TDRL) or in green representing transpositions. Genes colored in orange represent elements that moved to the right and in blue to the left. (a) Family diagram for M. senhousia; (b) Family diagram for P. viridis; (c) One transposition involving the movement of ND3 to the right and of the tRNA Tyr to the left; (d) Four TDLRs. [file peerj-10-13953-s001.pdf]

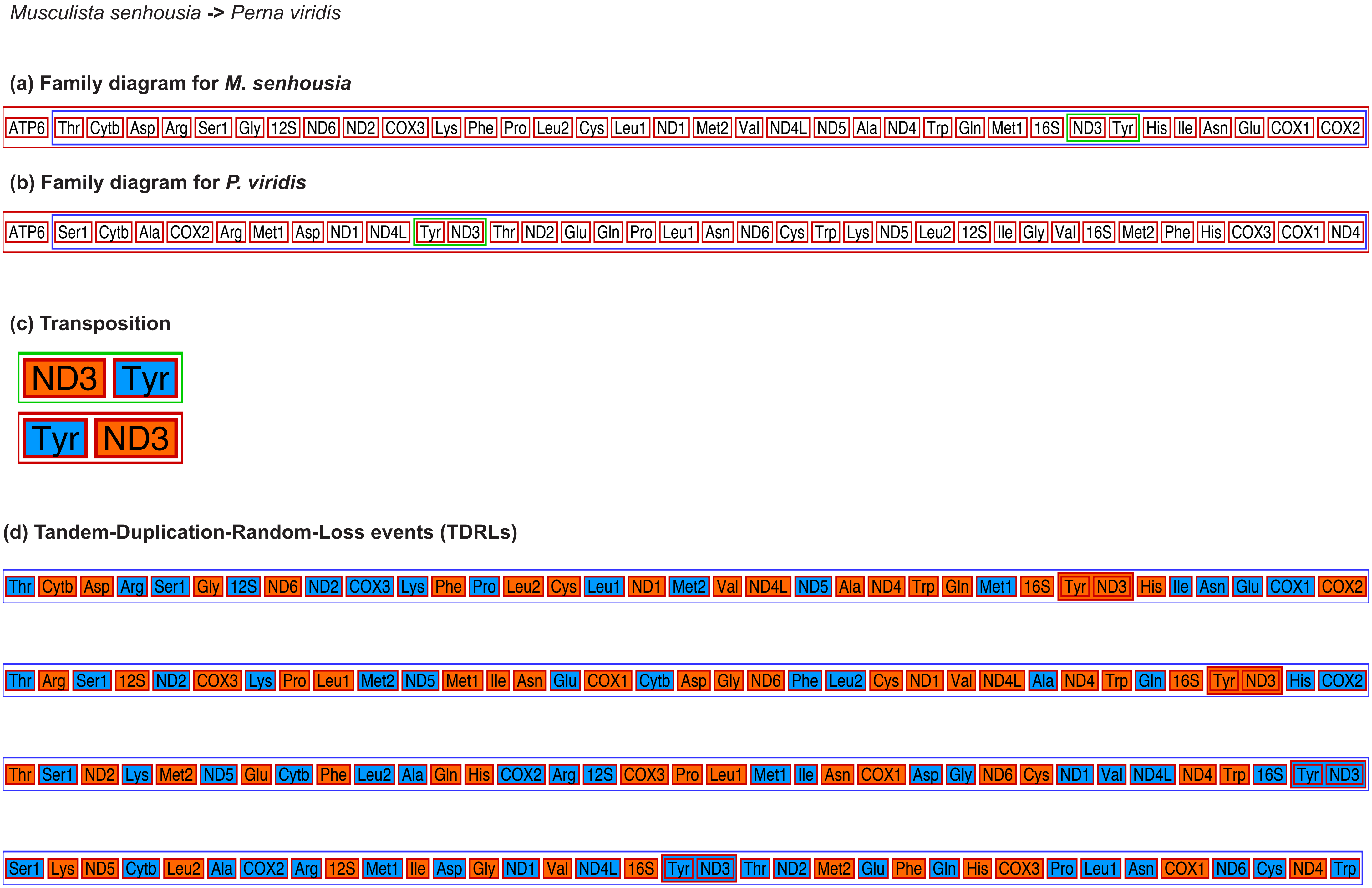
Musculista senhousia -> Perna viridis
(a) Family diagram for M. senhousia
ATP6 Thr Cytb Asp Arg Ser1 Gly 12S ND6 ND2 COX3 Lys Phe Pro Leu2 Cys Leu1 ND1 Met2 Val ND4L ND5 Ala ND4 Trp Gln Met1 16S ND3 Tyr His Ile Asn Glu COX1 COX2
(b) Family diagram for P. viridis
ATP6 Ser1 Cytb Ala COX2 Arg Met1 Asp ND1 ND4L Tyr ND3 Thr ND2 Glu Gln Pro Leu1 Asn ND6 Cys Trp Lys ND5 Leu2 12S Ile Gly Val 16S Met2 Phe His COX3 COX1 ND4
(c) Transposition
ND3 Tyr
Tyr ND3
(d) Tandem-Duplication-Random-Loss events (TDRLs)
Thr Cytb Asp Arg Ser1 Gly 12S ND6 ND2 COX3 Lys Phe Pro Leu2 Cys Leu1 ND1 Met2 Val ND4L ND5 Ala ND4 Trp Gln Met1 16S Tyr ND3 His Ile Asn Glu COX1 COX2
Thr Arg Ser1 12S ND2 COX3 Lys Pro Leu1 Met2 ND5 Met1 Ile Asn Glu COX1 Cytb Asp Gly ND6 Phe Leu2 Cys ND1 Val ND4L Ala ND4 Trp Gln 16S Tyr ND3 His COX2
Thr Ser1 ND2 Lys Met2 ND5 Glu Cytb Phe Leu2 Ala Gln His COX2 Arg 12S COX3 Pro Leu1 Met1 Ile Asn COX1 Asp Gly ND6 Cys ND1 Val ND4L ND4 Trp 16S Tyr ND3
Ser1 Lys ND5 Cytb Leu2 Ala COX2 Arg 12S Met1 Ile Asp Gly ND1 Val ND4L 16S Tyr ND3 Thr ND2 Met2 Glu Phe Gln His COX3 Pro Leu1 Asn COX1 ND6 Cys ND4 Trp
