# Supplementary material for: Comparative mitogenomic analyses and gene rearrangements reject the alleged polyphyly of a bivalve genus
Source: PeerJ. 2022 Sep 26;10:e13953. doi: 10.7717/peerj.13953 (PMC9521344; doi:10.7717/peerj.13953)

Perna viridis **->** *Perna canaliculus, P. perna* eastern and western SA, Brazil B2

(a) Family diagram for *P. Viridis*

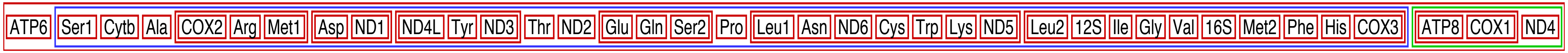

(b) Family diagram for *P. canaliculus, P. perna* eastern and western SA, Brazil B2

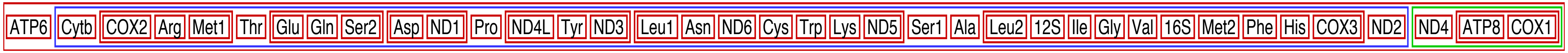

(c) Transposition

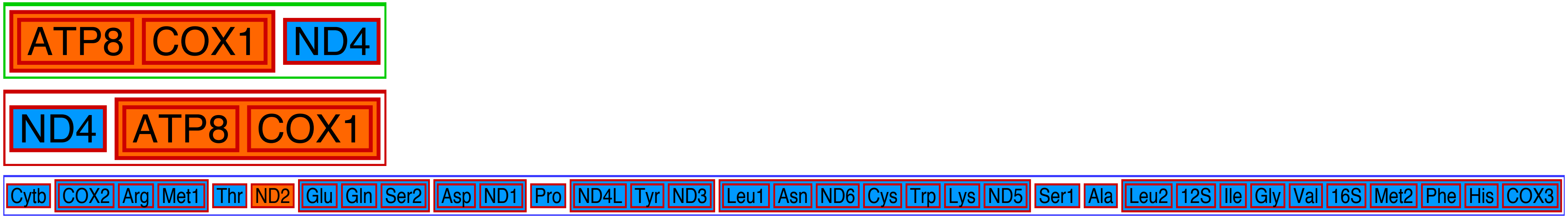

(d) Tandem-Duplication-Random-Loss events (TDRLs)

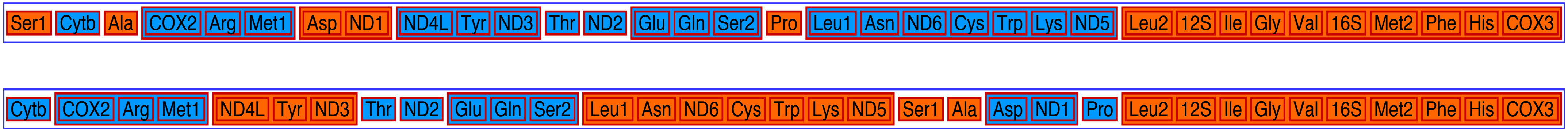

Supplement: Supplemental Information 2 — Gene rearrangement scenarios from the ancestral gene order of Perna viridis to the gene order of P. canaliculus/P. perna (eastern and western South Africa and Brazilian specimen B2) identified by the CREx analysis. Family diagrams represent the observed gene order and blocks of inferred rearrangement events highlighted in blue representing tandem-duplication-random-loss (TDRL) or in green representing transpositions. Genes colored in orange represent elements that moved to the right and in blue to the left. (a) Family Diagram for P. viridis; (b) Family diagram for P. canaliculus; (c) Transpositions involving the movement of the block [ATP8+COX1] and ND2 to the right and of ND4to the left; (d) Two TDLRs. [file peerj-10-13953-s002.pdf]
