# Supplementary material for: Comparative mitogenomic analyses and gene rearrangements reject the alleged polyphyly of a bivalve genus
Source: PeerJ. 2022 Sep 26;10:e13953. doi: 10.7717/peerj.13953 (PMC9521344; doi:10.7717/peerj.13953)

Musculista senhousia **->** *Perna perna* **B1**

(a) Family diagram for *M. senhousia*

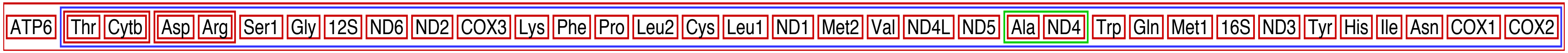

(b) Family diagram for *P. perna* B1

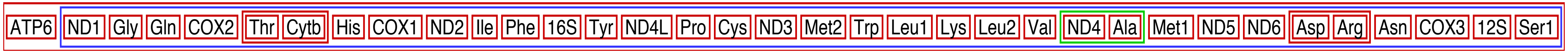

(c) Transposition

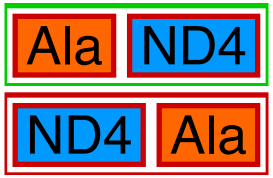

(d) Tandem-Duplication-Random-Loss events (TDRLs)

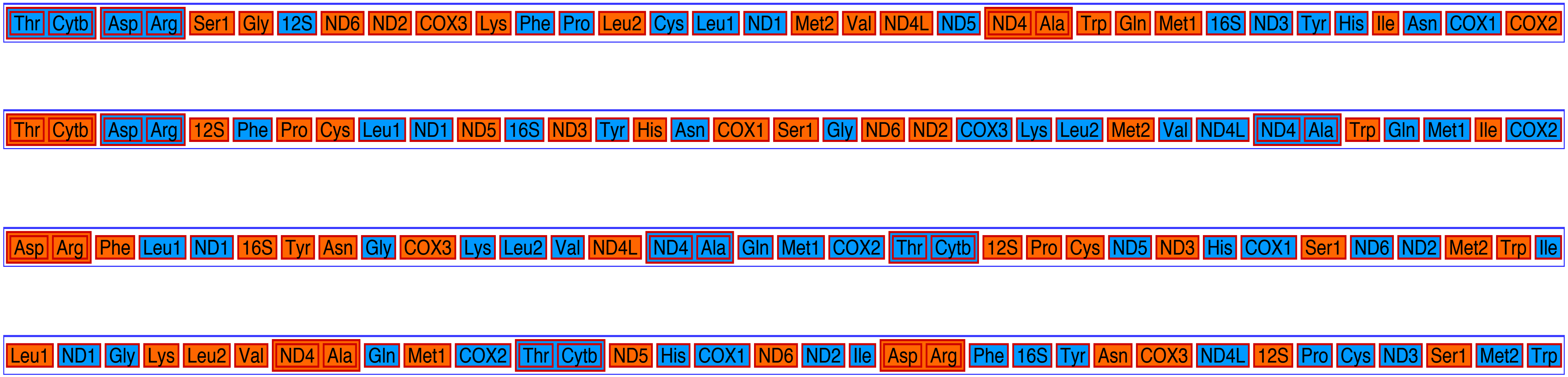

Supplement: Supplemental Information 4 — Gene rearrangement scenarios from the ancestral gene order of Musculista senhousia to the gene order of Perna perna (Brazilian specimen B1) identified by the CREx analysis. Family diagrams represent the observed gene order and blocks of inferred rearrangement events highlighted in blue representing tandem-duplication-random-loss (TDRL) or in green representing transpositions. Genes colored in orange represent elements that moved to the right and in blue to the left. (a) Family diagram for M. senhousia; (b) Family diagram for P. perna B1; (c) Transposition involving the movement of ND4L to the right and of the tRNA Tyr to the left; (d) Reversal; (e) Four TDLRs. [file peerj-10-13953-s004.pdf]
